# Within-female sperm allocation in *Enoploteuthis chunii* is associated with polyandry and male-biased sex ratio

**DOI:** 10.17912/micropub.biology.001538

**Published:** 2025-07-02

**Authors:** Md. Nur E. Alam, Anri Yamane, Satoshi Kusama, Noriyosi Sato, Noritaka Hirohashi, Hiroki Ono

**Affiliations:** 1 Graduate School of Natural Science and Technology, Shimane University, Matsue, Shimane, Japan; 2 Uozu Aquarium, Uozu City, Toyama, Japan; 3 Department of Fisheries, School of Marine Science and Technology, Tokai University, Tokyo, Tokyo, Japan; 4 Shimane University, Matsue, Shimane, Japan; 5 Oki MBS, Shimane University, Okinoshima, Shimane, Japan

## Abstract

One of the unusual reproductive features in cephalopods is the flexibility of sperm deposition sites in females. Recently we reported that female *Enoploteuthis chunii* possess not only a major sperm receptacle (MSR) but also a cryptic sperm pocket (CSP) and CSP utilization increases towards the end of the reproductive season. This may occur due to male-male competition over insemination sites, as a male-biased sex ratio and CSP utilization are tightly correlated. The microsatellite-based genotyping revealed that both MSR and CSP are used by the same males, a similar phenomenon previously found in *Loliolus sumatrensis* , which is herein hypothesized to be a strategy associated with sperm allocation.

**Figure 1. Males allocate their sperm investment to different storage sites within a female:**
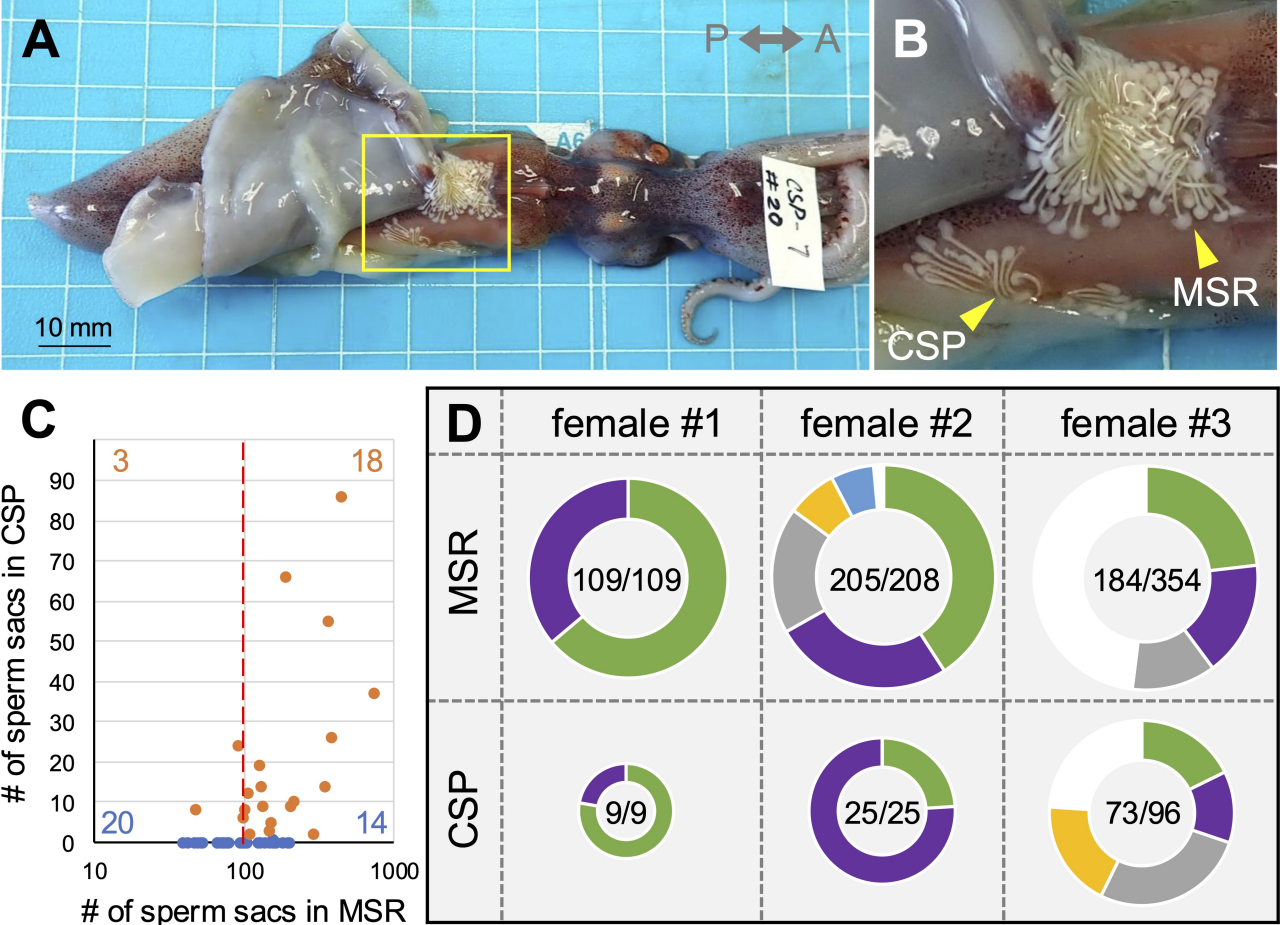
**A,** a dorsal view of a mated female with sperm sacs stored both in the MSR and the CSP. **B,** *inset* shows an enlarged image of the boxed area in A. **C,** the usage patterns of two sperm deposition sites (MSR and CSP) in female individuals. Most females that used the CSP, had more than 100 sperm sacs in the MSR. Females with ( *orange closed circles* ) or without ( *blue closed circles* ) sperm sacs in the CSP. Furthermore, the numbers of individuals divided by a threshold of 100 sperm sacs in the MSR ( *a broken red line* ) are shown in the corners. **D,** the pattern and number of sperm genotypes in three females that had sperm sacs in both the MSR and CSP. In each pie chart, "the total number of sperm sacs successfully genotyped / the total number of sperm sacs found attached" is displayed. Each color in the same female experiment represents an identical genotype.

## Description

In terms of reproductive physiology, sperm must successfully reach an egg (Yanagimachi, 1994). Additionally, in the context of intra-sexual competition, sperm must reach an egg faster than sperm from other males (Parker & Pizzari, 2010). Therefore, it is crucial for males to determine when, where and how much sperm should be deposited or inseminated in the female. Although males are commonly capable of producing a large amount of sperm, sperm is still considered a limited resource in the circumstances where intra-sexual competition is significant (Squires, 1979). The theory of sperm precedence predicts that male mating strategies have evolved through a selective force that favors obtaining greater female reproductive resources (eggs), and consequently more offspring of their own (Singh et al., 2002). There are primarily two modes of strategies: offensive and defensive. In the former, males remove, spoil or even kill preexisting sperm in the female (Silberglied et al., 1984; Harshman & Prout, 1994; Hayakawa et al., 2002). In the latter, males guard, prevent or discourage females from further copulations (Malouines, 2017; Stockley et al., 2020; Schausberger et al., 2023). The theory of sperm allocation predicts that males can manipulate the amount of sperm to deposit in response to reproductive conditions of mating females such as fecundity (Pitnick & Markow, 1994) and promiscuity (Gage & Barnard, 1996). Socio-sexual environments can also influence sperm allocation strategy.

In cephalopods, a wide range of mating behaviors and strategies have been documented, among which one of unusual reproductive features is the flexibility of sperm deposition sites within females (Marian et al., 2019; Sato, 2021). Insemination polymorphism occurs not only inter-specifically but also intra-specifically, i.e., there are two or more sperm deposition sites within a female (Hanlon & Messenger, 2018). Insemination dimorphism most commonly occurring in some Loliginidae squid species can be explained by alternative mating strategies where two or more discontinuous variations in mating behavior and morphology occur as the fitness consequence of male individual variability in physical competitiveness (Iwata et al., 2011). This suggests that the choice of insemination site by certain males is predetermined ontogenically or altered conditionally. Importantly, males choose only one of two sites in the female per copulation. On the contrary, in *Loliolus sumatrensis* , females possess three discontinuous insemination sites that are often used by the same males simultaneously (Azad et al., 2024). Although the adaptive significance of having three separate insemination sites remains unknown, it is possible that these sites are involved in sperm allocation, with each site providing a distinct benefit.

Our previous study on *Enoploteuthis chunii* revealed that females possess two distinct sperm deposition sites—one on the dorsal trunk around the posterior neck area inside the mantle cavity and the connected inner surface of the dorsal mantle, hereafter called the major seminal receptacle (MSR), and the other on the right lateral trunk within the inner mantle ( Fig. 1A, B; Moriwaki et al., 2024). The latter is located in a hidden place, and is therefore called a cryptic sperm pocket (CSP). We found that the CSP was utilized as a sperm reservoir only when more than 100 sperm sacs were found stored in the MSR, and the number of sperm sacs stored was consistently higher in the MSR than in the CSP ( Fig. 1C ). These results suggest that MSR was initially preferred, followed by the use of CSP. Next, we developed microsatellite markers (Methods, Extended Data file). Using these markers, we genotyped the sperm sacs that were found attached to the female sperm deposition sites. The results clearly demonstrated that the same males use both MSR and CSP to deposit their sperm sacs. We speculated that switching insemination sites during a single mating episode might have occurred. We also assume that sperm sacs in the CSP have limited accessibility from other males, making sperm displacement by subsequent males impractical. In fact, there are signs of sperm displacement (truncated spermatangia) in the MSR but not in the CSP (see Fig. 1G in Moriwaki et al., 2024). Taken together, we hypothesize that in this species, as the mating season nears its end and the sex ratio becomes male-biased, 1) the sperm allocation strategy prioritizes maximizing and securing sperm investment in a female even if fertilization opportunities are low, 2) and aternatively, the space available within the preferred site (MSR) gradually decreases. This could result in males having to transfer some of their sperm investment to a less favored site (CSP) during a single or consecutive mating episodes.

## Methods

Specimens 

Dead squids, E. chunii, were obtained as previously described (Moriwaki et al., 2024). Shortly, the squids were obtained as bycatch items in commercial white shrimp (Pasiphaea japonica) trawls at Toyama Bay off Iwase at depths between 150-300 m. Bi-weekly sampling was carried out from May to September 2021, totaling 12 days for the analysis used in this study. Additionally, specimens were collected in October and November 2021 (4 days) and August-October 2020 (5 days). 

Development of microsatellite markers 

Genomic DNAs were purified from testes (wet weight of ~ 20 mg) of 40 representative mature males with kits (QIAGEN Genomic-tip 20/G and TAKARA NucleoSpin Tissue) according to manufacture protocols, verified their degradation levels with 0.8% agarose gel electrophoresis followed by visualization with ethidium bromide, and quantified their yield and quality with a micro-volume spectrophotometer (NanoDrop One, Thermo Fisher Scientific) and stored at − 80 °C. Short-read whole genome sequencing (150 bp paired-end, Novaseq6000/PE150, Novogene) was carried out, which yielded a total of 24,841,624 clean reads that were thereafter merged with PEAR (https://cme.h-its.org/exelixis/web/software/pear/doc.html), resulting in 7,218,056 overlapped paired-end reads. The clean reads were registered in the DNA Data Bank of Japan (DDBJ) Sequence Read Archive under accession number: DRR629084 (Run), PRJDB19839 (BioProject) SAMD00855392 (BioSample) and DRX609412 (Experiment). Next, the sequence data were uploaded to Galaxy to search for short tandem repeat (STR) with a MISA + Primer 3 pipeline, which detected a total of 162,375 STRs. For an initial PCR test, 33 SSRs were selected at random and non-labelled primers synthesized. PCR was carried out using DNA polymerases (KAPA2G Robust HotStart ReadyMix and Platinum™ Direct PCR Universal Master Mix) and a thermal cycler (MiniAmp, Thermo Fisher Scientific) at optimized conditions: 20 ng of genomic DNA, 0.2 µM paired primers and PCR reaction consisting of an initial denaturing step of 94 °C for 2 min, then 40 cycles of 94 °C for 15 s, 56 °C for 15 s and 68 °C for 20 s followed by a final extension of 68 °C for 5 min. Amplicons with genomic DNAs from 10 males were subjected to run on 8% mini-slab polyacrylamide gel electrophoresis to verify apparent variability in size. Thereafter, four primer sets were selected to proceed fragment length analysis. Fluorescence (Hex, Fam, Cy3 and Ros)-tagged universal primers and target sequence-specific forward primers with tail sequences were used according to Blacket et al (Blacket et al., 2012). Fragment length analysis (ABI PRISM 3130xl Genetic Analyzer) was performed with GeneScan™ 600 LIZ dye Size Standard (Thermo Fisher Scientific). Subsequently, the four selected microsatellite loci were fully characterized by open-source tools, OSIRIS (National Institutes of Health) and GenAlEx v.6.5.1. Information and characterization of newly developed SSR markers are avairable in Extended Data. 

Genotyping of sperm sacs by fragment length analysis 

Dissection of sperm sacs (spermatangia) from three females were carried out as previously reported (Azad et al., 2024). Briefly, under a stereomicroscope, the female tissues that contain attached sperm sacs were dissected out and fixed in 70% ethanol for 1 h, thereafter every single spermatangium removed with fine forceps from the tissue was placed carefully into the bottom of each well of the 96-well plate. To each well, 10 µl of lysis buffer containing 0.1 mg/ml Proteinase-K (Direct PCR Master mix kit, Thermo Fisher Scientific) was added, followed by a 30-min incubation at 52 °C and heat (95 °C) inactivation for 1 min. PCR and fragment length analysis were performed as described above.

## Data Availability

Description: Newly developed microsatellite markers.. Resource Type: Dataset. DOI: https://doi.org/10.22002/e4h1y-fsp76
